# The Liver and Its Secretions

**Published:** 1852-06

**Authors:** Hanfield Jones


### SELECTIONS.

From the London Lancet.

*The Liver and its Secretions.* By Dr. HANFIELD JONES.

A short summary having been given of the points regarding the structure of the liver that are well ascertained, the three different opinions were noticed that have at present found supporters—first, that of Thiernan, Leidy, and Retzius, to the effect that the hepatic cells are contained in anastomosing tubes of basement membrane forming a plexus; secondly, that of N. Guillot, Genlach, and Carpenter, who believe that the tubular ducts cease at the margin of the lobules, but are continuous with a plexus of intercellular passages, extending throughout each lobule; thirdly, the one maintained by the author, that the ducts terminated by closed extremities, either even and rounded, or irregular, and do not expand to enclose the cells in a plexus, or proceed further as intercellular passages. A succinct account of the composition of the bile, taken from the work of Prof. Lehmann in Physiological Chemistry, next followed. The cholic, or cholalic acid is the fundamental organic acid in the biliary secretion; it is united with two other substances, glycocal and taurine, to form the conjugate acids, the glycocolic, and taurocholic, which are themselves again combined with soda, forming two salts. Choloidynic acid is isomeric to cholic acid, but contains no basic matter; it, as well as another substance, dyslycin, are decomposition products of cholic acid, from which they can also be produced by chemical means. The coloring matter of the bile presents itself in two principal varieties, named cholopyrrhin and bilivendin, the one, as its name imports, being yellow, the other green. The first is found in carnivorous animals and in man; the second in birds, fishes, and amphibia. The opinion of Schlossbergen, in opposition to that of Lehmann, was adopted by the author, with respect to the green color of the evacuations often passed by young children. Schlossbergen believing the color to depend on a modification of the bile, Lehmann on the presence of blood. The large quantity of mucus occurring in the bile was noticed, and a source pointed out for it, as well in the columnar particles of the epithelium as in the mucous glands, which Thiele discovered in the larger hepatic ducts. In discussing the question as to the formation of bile, the experiments of Hernde were noticed, who extirpated the livers of frogs, but did not find, as he had expected, that biliary matter had accumulated in their blood. From this fact, and from the circumstance that biliary matter could not be detected in portal blood, the conclusion was arrived at that the bile was formed in the liver, and not in the blood itself. The richness of the blood of the portal vein compared with that of the hepatic in fatty matter, the proportion being as 3.225 : 1.855, was quoted as rendering it very probable that the organic matters of the bile were formed out of the oily matter in the blood arriving at the liver. The other secretion of the liver, the glucose or sugar, was next adverted to, and the various facts mentioned which have been ascertained respecting it. It was particularly observed, that while sugar was readily detectible by Trommer's test in almost all livers, it appeared to be absent from the organ when it passed into a fatty condition. This had been observed in the human subject, in the fœtuses of animals, and in fishes. The relation of liver-sugar to the disease named diabetes, was next considered; and the theory was propounded, that the formation of sugar in the body, being a natural process, our aim, as physicians, should be, not to prevent it, but to animate and invigorate the vital functions, in the course of which this sugar is destroyed and eliminated from the system, probably as carbonic acid. Two cases were quoted, confirming this view—one from the practice of Dr. Hamilton Roe, and the other from that of Dr. Sieveking. The hypotheses of Schmidh and Lehmann, as to the relations that subsist between oily matter, bile, and liver-sugar, were next expounded. The former considers oily matter to break up into sugar and water, on the one hand, and cholic acid and water on the other, its two constituents, glycerine and oleic acid, with certain proportions of oxygen, being equivalent to sugar and cholic acid. The view of Lehmann, which considers cholic acid to result from the union of oleic acid and sugar, was preferred by the author, as allowing greater latitude to the bile-generating process. The remark of Lehmann, that the oil and oily acids contained in the bile are for the most part in a saponified state, and his deduction therefrom, that the hepatic cells did not evacuate their contents, wherein the oil occurs in a free, non-saponified state, by bursting, was remarked upon as confirming and extending the author's argument, that unless the ducts at and near their extremities exerted an active elaborating faculty, so as to form the bile out of oily matter, it was impossible to account for the presence of a gall-bladder, filled with healthy-looking bile, belonging to a liver which was no more than a mass of oil; nor how it occurred, that while the liver parenchyma contained abundance of sugar, none was found in the bile. A view, differing considerably from that commonly accepted, was now proposed, to the effect that the cells of the liver were not the constant and necessary agents in the secretion of bile; that the occurrence of this secretion in them was rather to be viewed as an accidental, if not a pathological phenomenon, the real function of the cells being to produce the sugar out of the blood supplied to them, which was then absorbed, and carried off by the hepatic vein. The arguments in support of this opinion were, that in perfectly healthy livers of animals, bile is not ordinarily to be seen unequivocally in the contents of the cells, while it is very common in those of persons who have died of various diseases; and that in extracts made of the parenchymata of various livers, Pettenkoffer's test gave very little or no evidence of the presence of biliary matter. Some other experiments were related, as to the action of various cholagogue medicines on the liver, which will elsewhere be more fully related.

Several of the members addressed the Society at the conclusion of Dr. Jones' paper; the remarks, however, were generally of an interrogatory character, and did not embrace anything which could be made available for a report. Indeed, the paper was rather of a descriptive and demonstrative character than a debateable one. The tests for the detection of sugar, &c., mentioned in the paper, were ably exemplified by Mr. Bligh.

